# Human gene-engineered calreticulin mutant stem cells recapitulate MPN hallmarks and identify targetable vulnerabilities

**DOI:** 10.1038/s41375-023-01848-6

**Published:** 2023-02-22

**Authors:** Johannes Foßelteder, Gabriel Pabst, Tommaso Sconocchia, Angelika Schlacher, Lisa Auinger, Karl Kashofer, Christine Beham-Schmid, Slave Trajanoski, Claudia Waskow, Wolfgang Schöll, Heinz Sill, Armin Zebisch, Albert Wölfler, Daniel Thomas, Andreas Reinisch

**Affiliations:** 1 Department of Internal Medicine, Division of Hematology, Medical University of Graz, Graz, Austria; 2 Research Institute of Molecular Pathology (IMP), Vienna BioCenter (VBC), Vienna, Austria; 3 Vienna BioCenter PhD Program, Doctoral School of the University of Vienna and Medical University of Vienna, Vienna BioCenter (VBC), Vienna, Austria; 4 Diagnostic & Research Institute of Pathology, Medical University of Graz, Graz, Austria; 5 Core Facility Computational Bioanalytics, Medical University of Graz, Graz, Austria; 6 Leibniz Institute on Aging, Fritz Lipmann Institute, Jena, Germany; 7 Institute of Biochemistry and Biophysics, Faculty of Biological Sciences, Friedrich-Schiller-University, Jena, Germany; 8 Department of Obstetrics and Gynecology, Medical University of Graz, Graz, Austria; 9 Otto Loewi Research Center for Vascular Biology, Immunology and Inflammation, Division of Pharmacology, Medical University of Graz, Graz, Austria; 10 Cancer Program, Precision Medicine Theme, South Australian Health and Medical Research Institute (SAHMRI), Adelaide, Australia; 11 Adelaide Medical School, The University of Adelaide, Adelaide, Australia; 12 Department of Blood Group Serology and Transfusion Medicine, Medical University of Graz, Graz, Austria

**Keywords:** Myeloproliferative disease, Myeloproliferative disease, Haematopoietic stem cells, Cancer models

## Abstract

Calreticulin (*CALR*) mutations present the main oncogenic drivers in *JAK2* wildtype (WT) myeloproliferative neoplasms (MPN), including essential thrombocythemia and myelofibrosis, where mutant (MUT) *CALR* is increasingly recognized as a suitable mutation-specific drug target. However, our current understanding of its mechanism-of-action is derived from mouse models or immortalized cell lines, where cross-species differences, ectopic over-expression and lack of disease penetrance are hampering translational research. Here, we describe the first human gene-engineered model of *CALR* MUT MPN using a CRISPR/Cas9 and adeno-associated viral vector-mediated knock-in strategy in primary human hematopoietic stem and progenitor cells (HSPCs) to establish a reproducible and trackable phenotype in vitro and in xenografted mice. Our humanized model recapitulates many disease hallmarks: thrombopoietin-independent megakaryopoiesis, myeloid-lineage skewing, splenomegaly, bone marrow fibrosis, and expansion of megakaryocyte-primed CD41^+^ progenitors. Strikingly, introduction of *CALR* mutations enforced early reprogramming of human HSPCs and the induction of an endoplasmic reticulum stress response. The observed compensatory upregulation of chaperones revealed novel mutation-specific vulnerabilities with preferential sensitivity of *CALR* mutant cells to inhibition of the BiP chaperone and the proteasome. Overall, our humanized model improves purely murine models and provides a readily usable basis for testing of novel therapeutic strategies in a human setting.

## Introduction

Myeloproliferative neoplasms (MPNs) are a group of clonal hematological stem cell disorders with an aberrant increase in mature myeloid lineages. The three Philadelphia chromosome (Ph)-negative subtypes are characterized by an excess of red blood cells (polycythemia vera, PV), platelets (essential thrombocythemia, ET) or by the deposition of reticulin fibers in the bone marrow (BM), termed myelofibrosis (MF) [1, 2]. Ph-negative MPNs are fueled by oncogenic driver mutations in either *JAK2*, *MPL,* or *CALR*, that are acquired in the hematopoietic stem and progenitor cell (HSPC) compartment [3–7]. While the *JAK2*^*V617F*^ mutation is detected in >90% of PV and 50–60% of ET and MF cases, the majority (70–80%) of *JAK2* non-mutated ET and MF patients harbor recurrent *CALR* mutations [6, 8]. More than 50 different mutations have been discovered in exon 9, however the two most common mutations, a 52-bp deletion (p.L367fs*46, DEL, type 1) and a 5-bp insertion (p.K385fs*47, INS, type 2) account for >80% of cases [9, 10]. Both mutation types cause a + 1-bp frameshift resulting in extended translation forming a novel c-terminal domain losing the KDEL endoplasmic reticulum (ER) retention motive while gaining positively charged amino acids [7]. Naturally, *CALR* is a highly abundant ER-resident calcium-sequestering chaperone, mediating nascent protein folding [11–13].

Recent reports demonstrated that mutant (MUT) *CALR* is capable of exiting the ER and binding to the thrombopoietin-receptor (TpoR, *MPL*), causing constitutive cytokine-independent activation of downstream signaling cascades which is considered the primary oncogenic mechanism of *CALR* mutations [14]. In established cytokine-dependent cell lines, expression of *CALR* MUT leads to cytokine-independent growth only when co-expressed with human *MPL* [15]. N-terminal glycan binding sites and homo-multimer formation via the novel c-terminal end of *CALR* MUT are required for *MPL* binding and activation [16–19]. However, little is known regarding the action of *CALR* MUT on human primary HSPCs and megakaryopoiesis and even less regarding its effect on the ER. Recently, cell line-based studies suggest N-glycosylation and activated IRE1α/XBP1 pathway as therapeutic vulnerabilities [20, 21].

In addition, several mouse models have been developed to clarify disease origins and mechanisms of *CALR* mutations [22]. These have shown that expression of the human type 1 *CALR* MUT C-terminus in hematopoietic cells drives thrombocytosis and accompanied megakaryocyte hyperplasia in the BM. However, murine models do not generate robust reticulin fibrosis in the BM and no competitive advantage of mutant HSPCs was observed upon transplantation [23–28]. Furthermore, species-specific biological characteristics, such as a different *MPL* binding capacity of mutant *CALR*, hamper direct translation of discoveries made in mice to humans [29]. Therefore, it is important to investigate human *CALR* mutations in an appropriate human cellular system.

Here, we demonstrate a genome engineering system with targeted integration of type 1 and type 2 *CALR* mutations into healthy HSPCs at their natural genomic loci, preserving endogenous expression [30–32]. This sophisticated system allows us to generate *CALR* MUT human HSPCs and to investigate the impact of mutation acquisition on HSPC phenotype, growth and differentiation properties. Additionally, we can investigate mutation-induced molecular consequences at the multipotent stem and progenitor level without confounding factors attributed to the heterogeneity of MPN patient material. Importantly, transplantation of engineered human *CALR* MUT HSPCs into immune-compromised mice recapitulates MPN hallmarks and enables investigation of early disease-initiating mechanisms in vivo. We demonstrated that this preclinical model can be readily used to discover novel mechanisms contributing to MPN pathology and to identify *CALR* MUT specific vulnerabilities for developing novel effective therapies in MPNs.

## Methods

### Primary cell isolation and cell culture

Umbilical cord blood and peripheral blood of MPN patients were collected following donor consent and institutional review board approval. CD34^+^ cells were enriched via magnetic beads (Miltenyi Biotech, Bergisch Gladbach, Germany) and flow sorting, respectively. CD34^+^ cells were cultured in StemSpan SFEMII (StemCell Technologies, Vancouver, BC, Canada) supplemented with human recombinant SCF, TPO, FLT3L, IL-6 (all 100 ng/ml, Peprotech, Rocky Hill, NJ, USA), StemReginin 1 (750 nM, Peprotech) and UM171 (35 nM, StemCell Technologies). TF-1_TpoR cells were cultured in RPMI1640 supplemented with 10% FBS and 10 ng/ml TPO.

### Single guide RNA (sgRNA) design

The *CALR* sgRNA was designed to intron 7. Target sequence: 5′-CGCCTGTAATCCTCGCCCAG-3′; sgRNA was acquired from Synthego (Redwood City, CA, USA) with chemical modifications.

### AAV vector production

Codon optimized *CALR* mutant or wildtype cDNA spanning exons 8 and 9 flanked by homology arms were cloned in the pAAV-MCS plasmid (Agilent #240071, Santa Clara, CA, USA). Vectors also included a fluorescent reporter gene (GFP, BFP or mCherry) under control of an SFFV-promoter downstream of the *CALR* cDNA. Recombinant AAV6 was produced as described previously [32].

### Electroporation and transduction

CD34^+^ HSPCs were electroporated 72 h after culture initiating using the Lonza 4D Nucleofection system and the Human Primary Cell Nucleofection Kit P3 (Lonza, Basel, Switzerland) with the following conditions: 1 × 10^7^ cells/ml, 150 µg/ml Cas9 protein (IDT, Coralville, IA, USA) pre-complexed with sgRNA at 1:2.5 molar ratio, program DZ-100. Following electroporation, cells were immediately transduced with 5000–10,000 vector genomes/cell of AAV6. Culture media was refreshed after 6–8 h and cells with high reporter expression were sorted on an Aria IIIu cell sorter (BD Biosciences, San Jose, CA, USA) 48 h post transduction.

### Colony assays

Sorted CRISPR-modified HSPCs were seeded either in methylcellulose media (MethoCult™ Enriched, triplicates, 300 cells each) or collagen-based media w/or w/o 50 ng/ml TPO (MegaCult™, quadruplicates, 1800 cells each, both StemCell Technologies) and incubated for 12–14 days. Collagen embedded colonies were fixed and stained magenta using a mouse anti-CD41 primary antibody (Biolegend, San Diego, CA, USA) followed by an anti-mouse alkaline phosphatase (AP) secondary antibody and AP substrate (Vector Laboratories, Burlingame, CA, USA). Colonies were counted and scored based on morphology and staining.

### Megakaryocyte differentiation

1.5 × 10^4^ modified HSPCs were cultured in 200 µl StemSpan SFEMII supplemented with 1 ng/ml SCF, 5 ng/ml TPO, 10 ng/ml IL-6, 10 ng/ml IL-9 and 0.4% LDL (triplicates). On days 4, 7, and 10, cells were stained with CD41-APC-Cy7 and CD42b-APC antibodies (BioLegend), mixed with counting beads (CountBright, Thermo Fisher Scientific, Waltham, MA, USA) and measured on a flow cytometer (CytoFLEX S, Beckman Coulter, Brea, CA, USA).

### Xenotransplantations

All mouse experiments were approved by the Austrian Ministry of Education, Science and Research (BMBWF-66.010/0056-V/3b/2019). 5 × 10^5^ CRISPR-modified HSPCs were intrafemorally transplanted into irradiated 8–12-week-old NSG mice. For competitive transplants, 1 × 10^5^ sort-purified HSPCs (1:1 mixture of *CALR* INS and WT cells) were intrahepatically transplanted into new-born NSGW41 mice. Human engraftment in murine bone marrow was evaluated after 8, 16, and 24 weeks via flow cytometry. Cells were stained with mTer119-BUV661, mCD45-APC-Cy7, hCD45-BB700, CD33-PE (BD Biosciences), CD19-SB600 (eBioscience, San Diego, CA, USA), CD41-PE-Cy7 (BioLegend) antibodies and SYTOX Red (Invitrogen, Carlsbad, CA, USA) and measured on a CytoFLEX LX flow cytometer (Beckman Coulter).

### RNA sequencing

2 × 10^5^ sort-purified CRISPR-modified HSPCs from three cord blood donors were cultured for two days in stem cell retention media before isolation of RNA (Monarch Total RNA Miniprep Kit, NEB, Ipswich, MA, USA). RNA-seq libraries were prepared with the TruSeq Stranded mRNA LT sample preparation kit and sequenced on a HiSeq 3000 instrument (Illumina, San Diego, CA, USA). Raw sequencing data was deposited in NCBI’s Gene Expression Omnibus (GSE195705). NGS reads were mapped using STAR and differential gene expression was calculated with DESeq2. Further data exploration was performed using GSEA (v.4.1.0), Enrichr (maayanlab.cloud/Enrichr) and STRING (v11.5).

### Inhibitor treatments

Sort-purified HSPCs and pre-diluted inhibitors were mixed and seeded with either methylcellulose media w/o TPO (triplicates) or collagen-based media with 50 ng/ml TPO (quadruplicates). Colonies were counted after 12–14 days and normalized to a DMSO treatment control. Bortezomib and HA15 were dissolved and diluted in DMSO, and final concentrations were 2 nM and 5 µM, respectively. Both inhibitors were purchased from MedChemExpress (Monmouth Junction, NJ, USA).

### Statistical analyses

All statistical analyses were performed using Graphpad PRISM 9 (Graphpad Software, San Diego, CA, USA). One-way ANOVA with Dunnett’s multiple comparison correction was used to test for statistical significance on bar graphs of in vitro experiments and two-way ANOVA on line graphs of megakaryocyte differentiation in liquid culture. Statistical significance of in vivo studies was evaluated using an un-paired student’s t-test (two-sided) with Welch’s correction or two-way ANOVA for repeated comparisons of subsequent time points. Detailed methods are available in the supplemental data.

## Results

### Generation of *CALR* MUT human HSPCs via CRISPR/Cas9 and AAV6-mediated site-specific knock-in

**Fig. 1:**
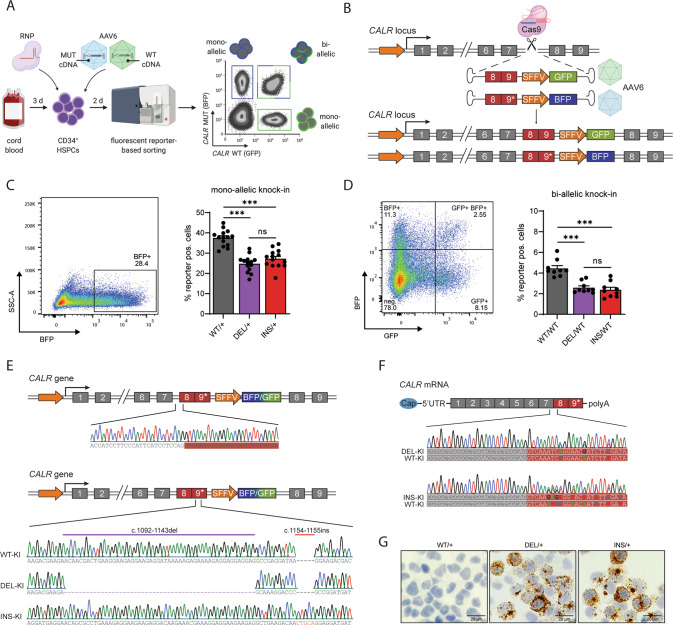
Generation of *CALR* MUT human HSPCs via CRISPR/Cas9 and AAV6-mediated site-specific knock-in. **A** Schematic workflow for introducing *CALR* MUT or WT cDNA into cord blood-derived CD34^+^ HSPCs using ribonucleoprotein (RNP) and recombinant adeno-associated virus serotype 6 (rAAV6). Fluorescent reporter-based sorting can be used to isolate cells with mono- or bi-allelic knock-in. Created with BioRender.com. **B** Detailed illustration of CRISPR/Cas9-mediated integration of mutant (*) or wildtype *CALR* cDNA via homology-directed repair (HDR). Inclusion of a downstream fluorescent reporter cassette (GFP/BFP) driven by a spleen focus forming virus (SFFV) promoter allows for tracking and enrichment of correctly edited cells. Gray boxes represent endogenous exons and red boxes exons from integrated cDNA harboring either WT or MUT sequences in exon 9. Bar graphs and representative flow plots with gating of fluorescent reporter positive cells show mono-allelic and bi-allelic *CALR* knock-in efficiencies in human HSPCs using a single (**C**) or dual (**D**) AAV6 approach. **E** Seamless 5’ integration confirms cDNA knock-in (top) with correct *CALR* WT, DEL and INS sequences in exon 9 (bottom). **F** Sequencing of reversely transcribed (RT) *CALR* mRNA shows correct splicing of endogenous exons to the integrated cDNA for each genotype. **G** Detection of *CALR* MUT protein in engineered HSPCs by immunocytochemistry using an antibody specifically detecting the mutation-specific novel C-terminus (CAL2, dianova). Staining within the endoplasmic reticulum (ER) indicated by brown speckles surrounding the nucleus. Bars represent mean ± standard error of mean (SEM) of 14 (single) and 9 (dual) independent biological replicates.

To introduce type 1 and type 2 *CALR* mutations at the endogenous gene locus in human HSPCs, we adopted our previously established, highly active knock-in (KI) strategy using CRISPR/Cas9 and recombinant adeno-associated virus serotype 6 (rAAV6) [30, 33]. CRISPR/Cas9 ribonucleoprotein (RNP), targeting intron 7 of *CALR* (upstream of the mutational hotspots in exon 9), and rAAV6 carrying a *CALR* MUT or WT cDNA donor template flanked with homology arms (Supplementary Fig. 1A), were co-delivered to primary human CD34^+^ HSPCs. Importantly, a SFFV-driven fluorescent reporter expression cassette (GFP or BFP) was integrated downstream of the cDNA to enable purification and tracking of modified cells via flow cytometry (Fig. 1A, B). We achieved stable on-target integration of *CALR* MUT and WT cDNA in 25–38% of HSPCs (DEL/+: 24.6 ± 4.4%, INS/+: 27.2 ± 4.4%, WT/+: 37.5 ± 4.0%, *n* = 14, Fig. 1C; Supplementary Fig. 1B). Since *CALR* mutations mainly occur heterozygously [6, 7], we aimed to guarantee this genotype by simultaneously integrating MUT and WT cDNAs coupled with distinct fluorescent reporters on individual alleles. This bi-allelic strategy resulted in 2.5–4.5% of heterozygous MUT HSPCs (DEL/WT: 2.58 ± 0.54%, INS/WT: 2.37 ± 0.74%, WT/WT: 4.45 ± 0.80%, *n* = 9, Fig. 1D; Supplementary Fig. 1C). Site-specific and seamless integration of *CALR* cDNA (Fig. 1E) and correct splicing of the exogenous cDNA to the endogenous upstream exons 1-7 resulting in a full-length *CALR* transcript (Fig. 1F) were confirmed for each genotype. Concomitant expression of WT and MUT transcripts indicated a heterozygous genotype. Abundant expression of *CALR* MUT protein was confirmed via immunocytochemistry (ICC) and Western Blot using a MUT-specific antibody targeting the novel c-terminal end. (Fig. 1G, Supplementary Fig. 1D, left panel). Total *CALR* protein levels were not altered, suggesting an intact cell intrinsic regulation of gene expression (Supplementary Fig. 1D, right panel). Collectively, these data indicate that our system can reliably generate primary human HSPCs carrying heterozygous *CALR* mutations for downstream mechanistic studies.

### *CALR* mutations induce TPO-independent growth and megakaryocyte differentiation in HSPCs

**Fig. 2:**
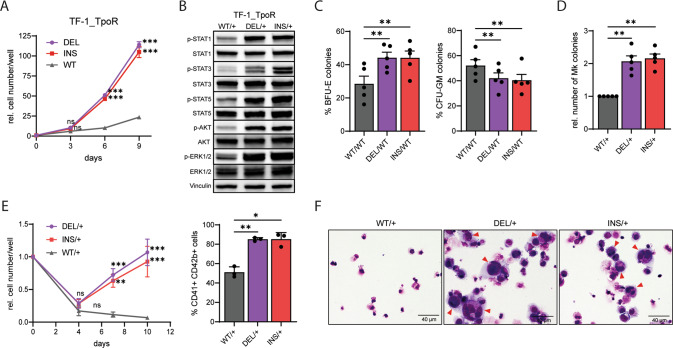
*CALR* mutations induce TPO-independent growth and megakaryocyte differentiation in HSPCs. TPO-independent proliferation (**A**) and increased TpoR-mediated downstream signaling (**B**) in TPO-deprived TF-1_TpoR cells confirms the functionality of mutant *CALR*. Differentiation potential of engineered HSPCs harboring heterozygous *CALR* mutations was assessed in methylcellulose-based colony forming unit (CFU) assays (**C**) and collagen-based megakaryocyte-specific CFU assays without TPO (**D**) with five independent biological replicates. Bar graphs show mean ± standard error of mean. BFU-E = Burst forming unit erythroid, CFU-GM = colony forming unit granulocyte-macrophage**. E** Proliferation (left) and megakaryocytic differentiation (right) of mono-allelic engineered HSPCs in low TPO (5 ng/ml) liquid culture (three independent biological replicates). Megakaryocyte differentiation was assessed by CD41 and CD42b cell surface marker expression at d10 of culture. **F** Pappenheim stainings of engineered cells at 10 days of liquid culture differentiation indicate formation of mature megakaryocytes (red arrows) with large multi-lobulated nuclei in *CALR* MUT cells only.

To functionally validate our KI strategy, we first introduced *CALR* mutations into thrombopoietin (TPO)-dependent TF-1 cells (TF-1_TpoR). Gene-engineered *CALR* MUT TF-1_TpoR cells showed TPO-independent proliferation (*p* < 0.001) (Fig. 2A) and increased phosphorylation of *MPL* downstream signaling mediators STAT1/3/5, AKT, and ERK1/2 in the absence of TPO (Fig. 2B). Since *CALR* MUT MPNs are characterized by altered differentiation of HSPCs, we performed methylcellulose-based colony-forming unit (CFU) assays to evaluate myeloid differentiation properties of engineered *CALR* MUT HSPCs. When compared to WT controls, both heterozygous *CALR* mutations induced a 1.5-fold increase (DEL/WT: 44.2 ± 7.5%, INS/WT: 44.1 ± 9.2% vs. WT/WT: 29.0 ± 11.9%) in erythroid (BFU-E) (p < 0.01) with a concurrent decrease (DEL/WT: 42.1 ± 9.5%, INS/WT: 40.5 ± 10.4% vs. WT/WT: 52.4 ± 12.1%) in myeloid (CFU-GM) colonies (*p* < 0.01) (Fig. 2C), suggesting a shift towards the megakaryocyte-erythroid lineage. On-target integration of *CALR* cDNAs was confirmed in individual colonies (Supplementary Fig. 2A). Since methylcellulose CFU assays do not support megakaryocyte (Mk) differentiation, we additionally performed a collagen-based CFU-megakaryocyte (CFU-Mk) assay [34]. In the presence of TPO (50 ng/ml), *CALR* MUT cells showed only a modest increase in Mk colonies (Supplementary Fig. 2B). However, under TPO-free conditions, we observed TPO-independent outgrowth of CFU-Mks from *CALR* MUT HSPCs, and only a limited number of small CFU-Mks derived from *CALR* WT HSPCs. Overall, *CALR* MUT HSPCs formed 2-fold (DEL: 2.1 ± 0.37-fold, INS: 2.2 ± 0.30-fold, *n* = 5) more CFU-Mk colonies than their WT counterparts (*p* < 0.01) (Fig. 2D). Similar results were obtained upon Mk differentiation in liquid-culture. *CALR* MUT cells showed significantly better survival (*p* < 0.001) in low TPO (5 ng/ml) conditions, became TPO-independent after 4 days, and formed more Mks expressing CD41 and CD42b (Fig. 2E). High TPO concentrations (50 ng/ml) however, resulted in efficient but unchanged differentiation (CD41 and CD42b expression) between genotypes but increased proliferation of INS cells (Supplementary Fig. 2C, D). Maturation of functional Mks was supported by the presence of multilobulated large nuclei and the formation of cellular protrusions indicating platelet shedding (Fig. 2F and Supplementary Fig. 2E).

### Genome-engineered human *CALR* MUT HSPCs show robust engraftment and induce myelofibrosis and splenomegaly in mice

**Fig. 3:**
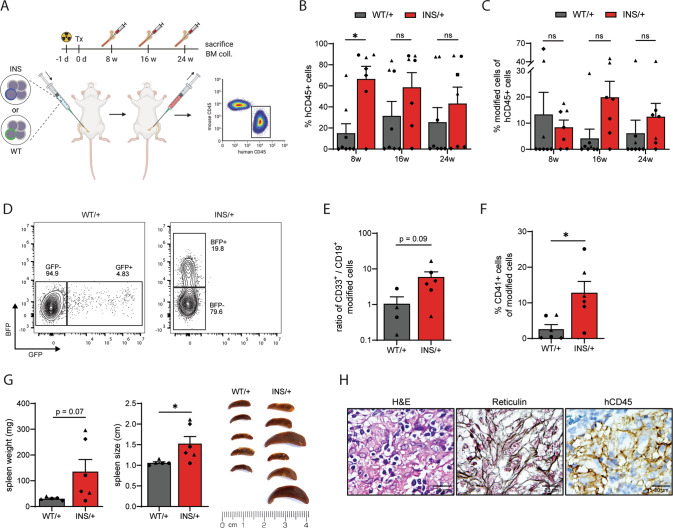
Genome-engineered human *CALR* MUT HSPCs show robust engraftment and induce myelofibrosis and splenomegaly in mice. **A** Schematic workflow of in vivo experiments. 5 × 10^5^ human CD34^+^ HSPCs, containing a sub-fraction of cells with *CALR* WT/+ or *CALR* INS/+ knock-in, were intrafemorally transplanted into 8–12 week-old sub-lethally irradiated (100 rad) NSG mice. HSPCs were derived from three independent cord blood donors and each donor was engineered to express *CALR* WT/+ and INS/+. FACS-based enrichment prior to transplantation was avoided to reduce cellular stress. Consecutive BM aspirations were performed to evaluate human engraftment before mice were sacrificed after 24 weeks. Created with BioRender.com. **B**, **C** Percentage (mean ± SEM) of total human CD45^+^ engrafted cells (**B**) and modified *CALR* INS or WT cells within CD45^+^ cells was assessed at 8, 16, and 24 weeks post-transplant (Tx) via flow cytometric detection of co-inserted fluorescent reporter expression (BFP or GFP). *N* = 7 mice. **(D)** Representative flow plots depicting fluorescence reporter positive cells within pre-gated total engrafted hCD45^+^ cells. **E**, **F** Contribution of engrafted engineered HSPCs to myeloid (CD33^+^), lymphoid (CD19^+^) and megakaryocytic (CD41^+^) lineage. In **E** CD33^+^/CD19^+^ ratio is depicted. **G** Weight (left graph) and size (right graph) of spleens from primary recipient mice measured at time of sacrifice (24 weeks post-Tx). **H** Representative images of H&E (morphology), Gomori (reticulin fibers) and hCD45 stained femur sections (left to right) from one mouse presenting excessive splenomegaly 24 weeks after receiving *CALR* INS HSPCs. Symbols (●, ▲, ♦) are used to label individual donors in all bar graphs depicted.

Efforts to generate MPN xenotransplantation models have been largely unsuccessful due to poor engraftment potential of MPN disease-initiating cells [35–37]. Here, we tested if our engineered *CALR* MUT human HSPCs retain repopulating capacity in immune-compromised mice. Therefore, *CALR* INS and WT HSPCs (*n* = 3 independent HSPC donors; each donor was engineered to express INS and WT to account for inter-donor variability) were transplanted intrafemorally into sub-lethally irradiated NSG mice (at least 2 mice per donor and group) and human engraftment was analyzed after 8, 16, and 24 weeks (Fig. 3A). Our model uniquely allowed tracking of modified cells in vivo over time by analyzing expression of the co-integrated fluorescent protein (GFP or BFP, Fig. 3C, D). Editing efficiencies prior to transplantation (Tx) were 40.2 ± 13.2% and 25.7 ± 7.2% for WT and INS respectively (Supplementary Fig. 3A). Mice receiving *CALR* INS HSPCs showed higher human engraftment (hCD45^+^ cells in murine BM) compared to WT HSPC recipients (Fig. 3B). Interestingly, upon successful engraftment, *CALR* INS cells outcompeted unmodified cells as evidenced by a 2.4-fold increase of BFP^+^ within hCD45^+^ cells between 8 (8.4 ± 2.8%) and 16 weeks (19.9 ± 6.1%) (Fig. 3C). A similar outgrowth advantage of *CALR* INS versus WT cells was seen in competitive Tx experiments using sort-purified cells, mixed at a 1:1 ratio prior to intrahepatic Tx into newborn NSGW41 mice (Supplementary Fig. 3D–F). In stark contrast, *CALR* WT HSPCs were regularly outperformed by the co-transplanted unmodified HSPCs between 8 (13.3 ± 8.4%) and 16 weeks (4.2 ± 3.5%) (Fig. 3C). Expression of *CALR* MUT protein was confirmed via ICC in engrafted hCD45^+^ BFP^+^ cells (Supplementary Fig. 3B). Engrafted *CALR* INS cells showed myeloid skewing with a high myeloid (CD33^+^) to lymphoid (CD19^+^) ratio (INS: 5.9 ± 2.3 vs. WT: 1.1 ± 0.6) and increased CD41^+^ Mk progenitor formation (INS: 12.7 ± 3.3% vs. WT: 2.6 ± 1.3%) (Fig. 3E, F). Strikingly, upon sacrificing, mice engrafted with *CALR* INS cells presented with increased spleen size (INS: 15.3 ± 1.7 mm vs. WT: 10.6 ± 0.2 mm) and weight (INS: 135.4 ± 47.3 mg vs. WT: 30.3 ± 2.6 mg). Two mice showed marked splenomegaly and clinical grade 2 reticulin fibrosis in the BM (Fig. 3G, H). Upon histological examination, spleens from both INS and WT transplanted mice showed engraftment of hCD45^+^ cells (Supplementary Fig. 3C). Secondary Tx revealed moderate conservation of stem cell and self-renewal properties of gene-engineered cells independent of *CALR* genotype as seen by low but detectable engraftment in secondary recipients (Supplementary Fig. 3G, H). This data shows that we can reproducibly generate a trackable and penetrant MPN phenotype on a heterozygous human *CALR* MUT background in vivo.

### *CALR* MUT human HSPCs show dysregulated ER stress response mechanisms

**Fig. 4:**
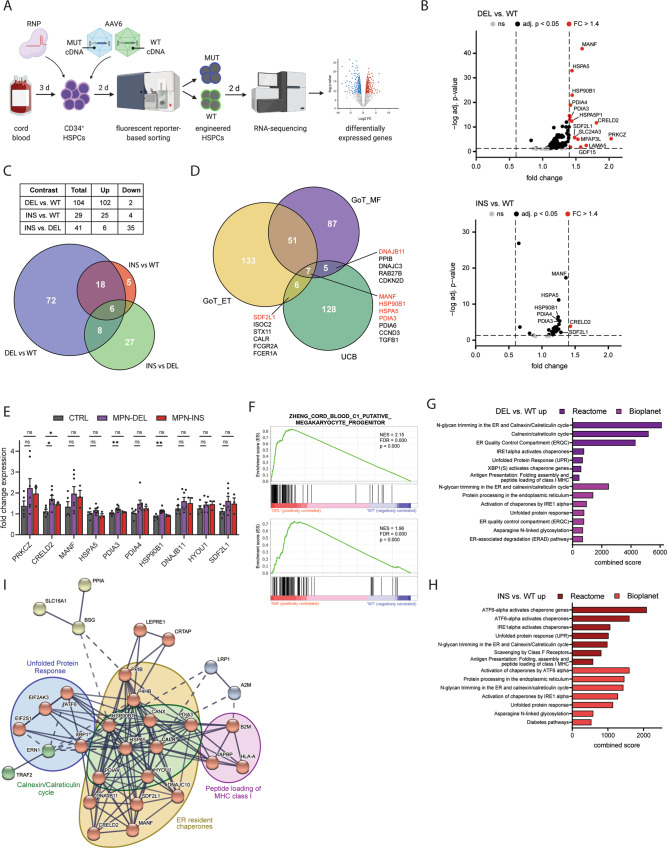
*CALR* MUT human HSPCs show dysregulated ER stress response mechanisms. **A** Schematic workflow showing cell preparation for RNA-sequencing. Human cord blood-derived CD34^+^ HSPCs were expanded for 3 days in vitro prior to CRISPR/Cas9-mediated knock-in of *CALR* MUT or WT cDNA. Reporter positive (GFP^+^ or BFP^+^) cells were purified using flow cytometry-based sorting and expanded for additional 2 days before isolation of RNA. *N* = 3 independent cord blood donors. Created with BioRender.com. **B** Volcano plots visualizing up- or down-regulated genes. Significantly deregulated genes (*p*_adj_ < 0.05) are shown in black and genes with FC > 1.4 were additionally highlighted in red. **C** Summary of up- and down-regulated genes comparing different genotypes. Venn diagram showing the overlap of differentially expressed genes (DEG) for each comparison. **D** Venn diagram showing the overlap of DEGs from our dataset with already published scRNA-seq data of ET and MF patient-derived CD34^+^ HSPCs [39]. **E** Differential expression (mean ± SEM) of the top ten DEGs in *CALR* MUT samples detected by RNA-seq was confirmed via RT-qPCR comparing CD34^+^ HSPCs from *CALR* MUT MPN patients (*n* = 10, 7× DEL, 3× INS) and healthy control HSPCs donors (CTRL, *n* = 5). **F** Representative enrichment plots determined by gene set enrichment analysis (GSEA) are shown for DEL (top) and INS (bottom) *CALR* mutations. Normalized enrichment score (NES), false discovery rate (FDR) as well as *p* value are reported alongside. **G, H** Enrichment analysis Enrichr webtool, maayanlab.cloud/Enrichr) of significantly upregulated genes (*p*_adj_ < 0.05) comparing DEL vs. WT (**F**) and INS vs. WT (**G**) was performed separately, and the top 7 enriched terms from Reactome and Bioplanet gene set databases are depicted. **I** Top ten commonly upregulated genes in *CALR* MUT versus WT were used to compute a protein interaction network diagram using STRING analysis. Bold lines indicate strong evidence and dotted lines weaker evidence for interaction. Node (protein) coloring is based on clustering via the MCL method. Known protein functions (colored and shaded circles) were manually annotated.

Activation of JAK-STAT signaling via binding of *CALR* MUT to TpoR is considered the key mechanism in *CALR*-driven MPN pathogenesis [16, 18, 38]. RNA-sequencing of engineered *CALR* MUT or WT human HSPCs (*n* = 3 independent donors) was performed four days after KI to ensure an immature HSPC state and allowed us to uncover important cellular mechanisms involved in early MPN development (Fig. 4A). Our data revealed a total of 104 (DEL vs. WT) and 29 (INS vs. WT) differentially expressed genes (DEGs) (*p*_adj_ < 0.05, Fig. 4B, C), with significant overlap between mutations and the vast majority of genes being upregulated in MUT cells. *CALR* INS cells additionally showed downregulation of 35 genes when compared to DEL, indicating expression changes that are unique to each mutation type. This was underlined by clustering of samples based on DEGs, where same genotypes clustered together with greater similarity between DEL and INS samples than to WT (Supplementary Fig. 4A, B). Confirming the validity of our data, we found that six of our top ten DEGs were overlapping with a previously published dataset, generated by single cell RNA-seq of ET and MF patient-derived CD34^+^ HSPCs [39] (Fig. 4D). We also confirmed upregulation of these top ten common DEGs in engineered *CALR* MUT HSPCs via RT-qPCR (Supplementary Fig. 4C) and in MPN-patient-derived CD34^+^ HSPCs (Supplementary Table 1) compared to healthy controls, with more pronounced effects seen in DEL than INS samples (Fig. 4E).

Gene set enrichment analysis (GSEA) was performed to discover cellular processes induced by *CALR* mutations. Our analyses revealed a strong enrichment for Mk progenitor associated signatures, both in DEL (NES = 2.15, *p* < 0.001, FDR < 0.001) and INS (NES = 1.96, *p* < 0.001, FDR < 0.001) cells (Fig. 4F). To get a closer view of induced pathways, we performed GSEA using Enrichr (maayanlab) with only significantly up-regulated genes in MUT cells as calculated by the DESeq2 package. DEL and INS cells showed a very similar enrichment profile with strong enrichment for ER stress-related signatures, including unfolded protein response (UPR)-mediated induction of chaperones. Furthermore, protein processing related signatures like calnexin/calreticulin cycle and N-glycan trimming were also enriched in *CALR* MUT cells (Fig. 4G, H). DEL MUT cells were also enriched for terms associated with platelet function and depleted for rRNA processing related terms (Supplementary Fig. 4D), whereas INS MUT cells showed strong enrichment for Golgi to ER retrograde transport and depletion of translation elongation (Supplementary Fig. 4E). These results were supported by a protein network analysis using STRING, revealing a highly interactive network with a core built by ER-resident chaperones, UPR mediators and proteins involved in peptide loading of MHC class I (Fig. 4I). Lastly, we elucidated gene expression differences between mutation types. INS compared to DEL cells were enriched for terms associated with cell proliferation including mRNA processing, separation of sister chromatids, and cholesterol biosynthesis (Supplementary Fig. 4F). Downregulated genes in INS cells were enriched for ATF6-mediated activation of chaperones and p53 signaling pathways (Supplementary Fig. 4G). Overall, our transcriptional profiling indicates that *CALR* mutations induce ER stress in immature HSPCs, leading to an UPR-mediated compensatory upregulation of chaperones and other protein processing pathways.

### Proteasome and BiP inhibition induce synthetic lethality in *CALR* MUT HSPCs

**Fig. 5:**
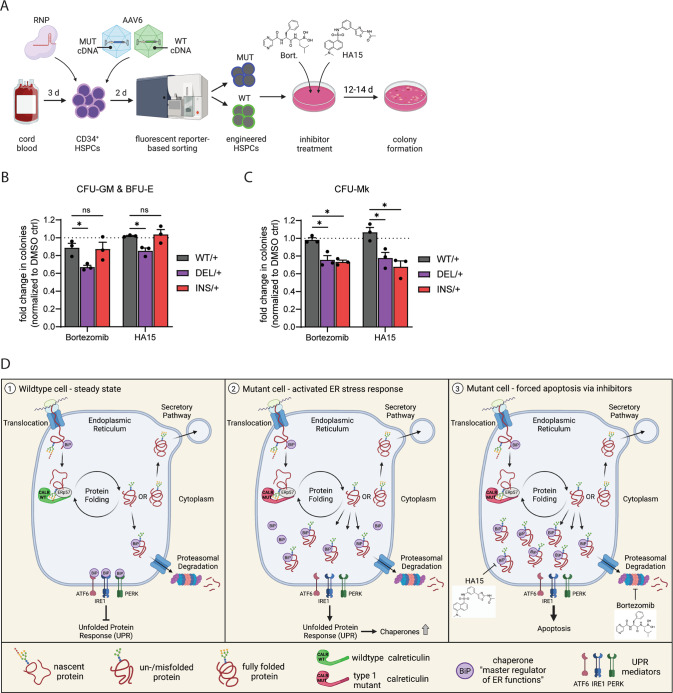
Proteasome and BiP inhibition induce synthetic lethality in *CALR* MUT HSPCs. **A** Schematic workflow of inhibitor experiments. Human cord blood-derived CD34^+^ HSPCs were expanded for 3 days prior to knock-in of *CALR* MUT or WT cDNA. Reporter positive cells were purified after 2 days using FACS and cells were seeded together with selected inhibitors into semisolid methylcellulose- or collagen-based media. The number of colonies formed was evaluated after 12-14 days. Created with BioRender.com. Fold changes (mean ± SEM) of combined CFU-GM and BFU-E colonies (**B**) and CFU-Mk colonies (**C**) normalized to DMSO controls after treatment with 2 nM Bortezomib and 5 µM HA15. *N* = 3 independent cord blood donors. **D** Graphical summary of the proposed mechanisms that are induced upon *CALR* mutation acquisition and upon selective targeting of vulnerabilities. *CALR* WT cells show steady state of proper protein folding and degradation of unfolded proteins (left panel). *CALR* MUT cells lack chaperone activity, therefore accumulate unfolded proteins which activates UPR to increase chaperone expression and compensate the lack in activity (center panel). Inhibition of BiP and the proteasome complex overloads cells with unfolded proteins and as a result activates apoptosis (right panel).

Based on the discovered compensatory upregulation of chaperones in *CALR* MUT HSPCs, we reasoned that direct inhibition of these chaperones or overloading of cells with unfolded proteins via proteosome inhibition, could be novel mutation-specific therapeutic vulnerabilities. To this end, we performed methylcellulose- (pan-myeloid, without TPO) and collagen-based (megakaryocytic, with TPO) CFU assays (Fig. 5A) with the selective BiP/HSPA5 inhibitor HA15 and the clinically approved proteasome inhibitor Bortezomib [40]. While Bortezomib and HA15 reduced CFU-GM and BFU-E colony numbers only of DEL mutant cells (HA15: 14.7 ± 3.2%, Bortezomib: 33.0 ± 2.1%, *p* < 0.05) (Fig. 5B), both drugs led to a significant reduction in CFU-Mk colonies in DEL as well as in INS mutant samples (HA15: DEL 22.3 ± 6.3%, INS 32.1 ± 6.6%; Bortezomib: DEL 24.3 ± 4.7%, INS 26.6 ± 2.0%, *p* < 0.05) (Fig. 5C). In summary, these data suggest that inhibition of cellular ER stress response mechanisms like chaperones and the proteasome complex provide novel strategies to selectively target *CALR* MUT MPNs (Fig. 5D).

## Discussion

In this study we describe the development of a novel *CALR* MUT MPN model, that for the first time allows a prospective investigation of human MPN development. We utilized a CRISPR/Cas9-mediated KI approach based on homology-directed repair (HDR) to insert the two most common *CALR* mutations into healthy primary human HSPCs [30, 33]. By preserving physiologic gene regulation, our site-specific KI at the endogenous *CALR* gene locus overcomes issues related to overexpression in retroviral delivery strategies [41]. Genome engineering via CRISPR/Cas9 and rAAV6 donors was shown to be feasible in primary human HSPCs [32, 42], however KI of genetic sequences remains challenging often resulting in low editing efficiencies [43]. Here, we describe a novel approach, concomitantly introducing either MUT or WT *CALR* sequences and a downstream fluorescent reporter cassette that allows us to i) purify gene-engineered cells using FACS and ii) track proliferation and engraftment of cells in vivo. A similar approach using TALENs and AAV6 has recently been described for the KI of *CD40L* cDNA in T-cells from patients with inherited X-linked hyper-immunoglobulin M syndrome [44]. However, whole length *CALR* cDNA (exons 1–9) exceeds the limited cargo capacity of rAAV6 (~4,7 kb). Therefore, we used cDNAs covering only exons 8 and 9, harboring type 1 and type 2 mutations. Importantly, by targeting intron 7 of *CALR*, we completely avoid the risk of disrupting the *CALR* reading frame through erroneous generation of insertions and deletions in cells without successful HDR events [45]. To guarantee correct splicing of the introduced cDNA to the upstream endogenous exons, we implemented a splice-acceptor sequence including the 3′ splice-site, branch point and the polypyrimidine tract (Supplementary Fig. 1A).

By prospectively assessing the functional consequences of *CALR* mutations on primary human HSPCs, our model reflected MPN hallmarks in vitro. Colony assays and liquid cultures showed megakaryocyte lineage skewing and acquisition of cytokine-independence, in line with recent reports from cell line models [16, 17, 46, 47]. Notably, *CALR* mutations do not immediately render HSPCs fully TPO-independent but provide growth advantages to megakaryocyte-primed progenitors. Interestingly, INS mutations seem to boost proliferation even in the presence of TPO pointing towards a TPO hypersensitive phenotype (Supplementary Fig. 2C). This is underscored by transcriptional data showing enrichment of proliferation-related terms in INS compared to DEL cells (Supplementary Fig. 4F). The presence of *MPL* was shown to be necessary for *CALR* MUT-induced aberrant JAK-STAT signaling [24]. However, the initial viability drop observed in our liquid-culture megakaryocyte-differentiation indicates that human HSPCs do not immediately gain a mutation-induced survival advantage via *MPL*-mediated signaling. The short latency necessary to induce cytokine-independence suggests additional transcriptional changes or external signals are required for the oncogenic transformation in HSPCs.

Recently, several studies reported the generation of heterozygous *CALR* MUT mouse models developing thrombocytosis and megakaryocyte hyperplasia in the BM [25–28]. However, other important disease hallmarks such as myelofibrosis and splenomegaly were only developed in mice with homozygous type 1 mutations, a genotype usually not found in MPN patients. In these homozygous models, myelofibrosis developed after a long latency of 6–10 months, whereas in our novel human heterozygous *CALR* MUT model, myelofibrosis was detected as early as 24 weeks post Tx. The low disease penetrance on a murine heterozygous background limits the translational impact of murine-only models. Although patient-derived xenotransplantation has the potential to overcome these hurdles, engraftment was restricted to MF samples so far, with less aggressive MPNs lacking meaningful engraftment levels [48, 49]. When transplanted into mice, our genome-engineered human *CALR* MUT HSPCs robustly engrafted and formed a reproducible and trackable humanized MPN model. We observed myeloid skewing, increase in BM megakaryocytes, and a competitive growth advantage of *CALR* MUT cells, in line with previous reports [48, 50]. Importantly, some engrafted mice developed BM fibrosis and splenomegaly. To our knowledge, this has not been demonstrated before on a heterozygous *CALR* MUT background, underlining the importance of studying human *CALR* mutations in the appropriate human cellular context. Human *CALR* MUT has a considerably greater binding affinity to human TpoR compared to murine TpoR. Enhanced activation of TpoR signaling in the transplanted human cells might therefore explain the emergence of myelofibrotic disease hallmarks even on a heterozygously mutant background [26]. The lack of statistical significance in some of our xenotransplantation experiments is a result of the high inter-donor engraftment variability. To overcome this issue, we preventively engineered HSPCs from each individual donor to express *CALR* WT and INS. Importantly, the phenotypic changes (splenomegaly, BM fibrosis) in our model were observed in mice transplanted with independent donors. One further limitation of xenotransplantation models is the known moderate self-renewal capacity of genome engineered HSPCs preventing propagation of disease phenotypes into secondary mice.

Expression profiling revealed early megakaryocyte priming in *CALR* MUT HSPCs, which is in line with recent reports from K562 cells and KI mice [51, 52]. This early priming might be an additional factor for the megakaryocyte skewing and expansion observed in the BM of our engrafted mice and in MPN patients. Besides the known *MPL*-dependent activation of JAK-STAT signaling, *CALR* mutations can induce additional mechanisms contributing to MPN development [53, 54]. *CALR* is known to be a key chaperone for the folding of N-glycosylated proteins within the ER [55–57]. N-glycosylation and UPR (IRE1α/XBP1 pathway) have been recently shown to be type 1 *CALR* MUT-specific therapeutic targets [20, 21]. Using our model, we similarly observed an upregulation of N-glycan trimming signatures and UPR-mediated induction of ER-resident chaperones in HSPCs, suggesting activation of a compensatory mechanism to sustain proliferation and cope with the accumulation of unfolded proteins induced by the loss of chaperone activity of mutant *CALR* (Fig. 5D). Targeting this compensatory mechanism in a direct fashion by inhibiting the key chaperone BiP or indirectly by inhibiting the proteasome and thereby preventing elimination of unfolded proteins presents a novel specific vulnerability for type 1 and type 2 *CALR* mutations. Both treatments are more effective in megakaryocyte-primed cells, possibly due to an enhanced *CALR* expression in these cells [58]. The proteasome inhibitor Bortezomib is already approved for the use in multiple myeloma [40] and would therefore present a promising candidate for immediate clinical translation of our findings. Nevertheless, further validation in MPN patient-derived cells and genetically engineered MPN mouse models is needed to fully evaluate the therapeutic potential of these inhibitors.

In summary, we reported a novel *CALR* MUT MPN model utilizing genome-engineered human HSPCs. Our engineered cells exhibit a megakaryocyte differentiation bias and cytokine-independent growth in vitro and recapitulate hallmarks of MPNs in vivo. Transcriptional profiling revealed mutation-induced ER stress responses like upregulation of compensatory chaperones and protein processing pathways as well as early megakaryocyte priming. Our findings provide unique insights into the molecular pathology of *CALR* MUT MPNs and enabled the identification of novel mutation-specific vulnerabilities via BiP and proteasome inhibition. In parallel, the accessible and reproducible in vivo MPN xenotransplantation model provides a readily usable basis for pre-clinical testing of novel therapeutic strategies in a human setting.

## Supplementary information

Fosselteder et al. revised supplementary material and figures

## Data Availability

RNA-Seq data are available at the GEO repository under accession number GSE195705. Original data generated in this study are available from the corresponding author on request.
