# Supplementary material for: Human gene-engineered calreticulin mutant stem cells recapitulate MPN hallmarks and identify targetable vulnerabilities
Source: Leukemia. 2023 Feb 22;37(4):843–53. doi: 10.1038/s41375-023-01848-6 (PMC10079532; doi:10.1038/s41375-023-01848-6)
Supplement: Supplementary file 1 — Fosselteder et al. revised supplementary material and figures [file 41375_2023_1848_MOESM1_ESM.docx]

# Supplementary Figures

­
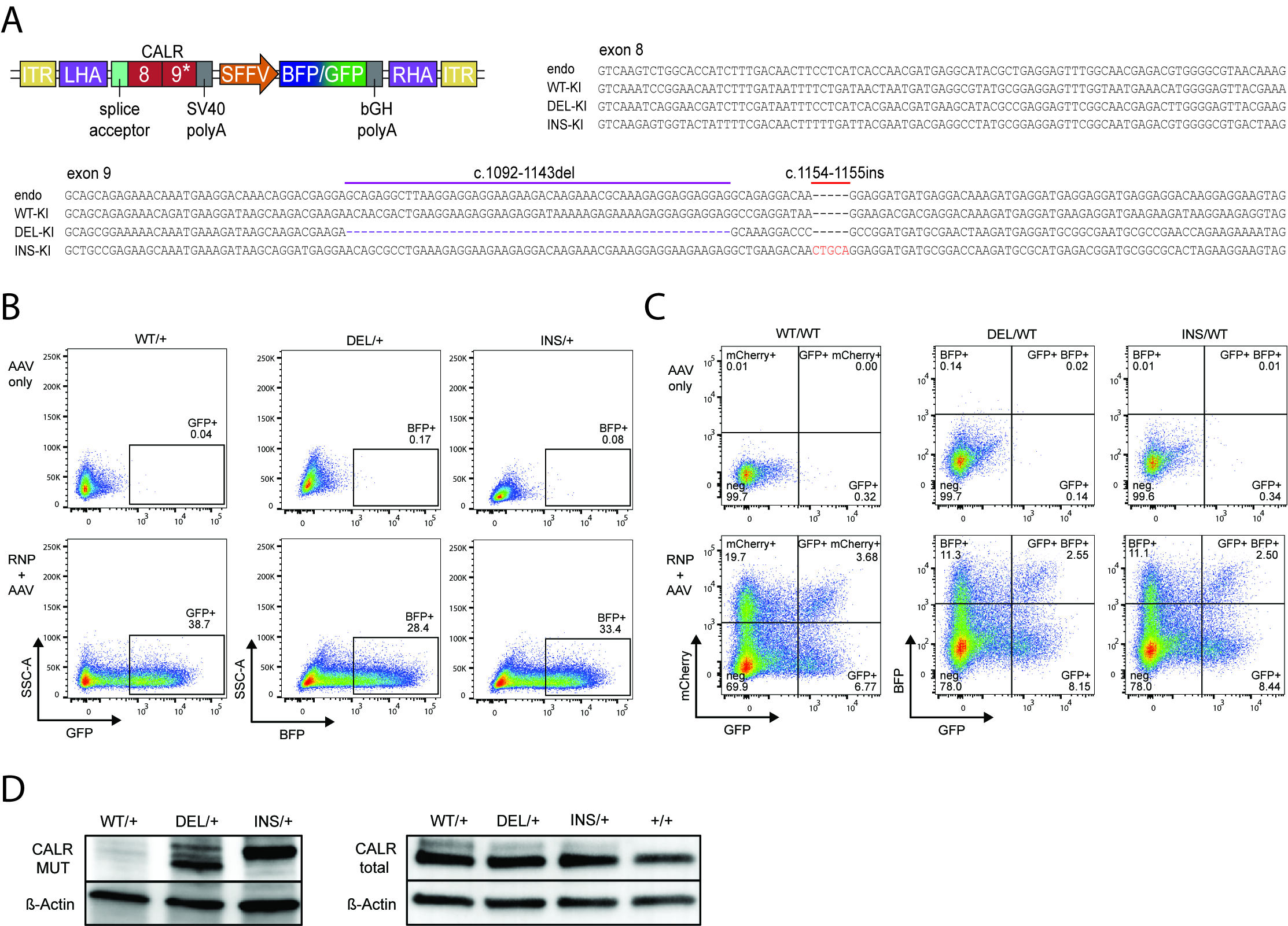


**Supplementary Figure 1. Generation of *CALR* MUT human HSPCs via CRISPR/Cas9 and AAV6 mediated site-specific knock-in. (A)** Schematic representation of rAAV6 donor DNA containing *CALR* cDNA (exon 8 and 9) with upstream splice acceptor from intron 7 and followed by a fluorescent reporter expression cassette driven by a SFFV promoter. Donor DNA sequences are flanked by homology arms (LHA/RHA) that are complementary to the double strand break (DSB) DNA ends in intron 7 and by AAV2 inverted tandem repeat (ITR) sequences. Endogenous and codon optimized knock-in cDNA sequences of *CALR* exon 8 and 9 are depicted for each genotype. (**B-C**) Representative flow plots from mono- (B) and bi-allelic (C) knock-in of WT (GFP; GFP/mCherry) or MUT (BFP; BFP/GFP) *CALR* cDNAs in human HSPCs. Reporter single or double positive cells were sorted for downstream assays respectively. AAV only transduced cells without knock-in were used to set sorting gates based on background signal of fluorescent reporters. **(D)** Western Blot analysis confirms expression of *CALR* MUT protein in genome-engineered HSPCs (left panel) and shows comparable total *CALR* protein expression between WT (WT/+), MUT (INS/+; DEL/+) and unmodified (+/+) HSPCs (right panel).

**
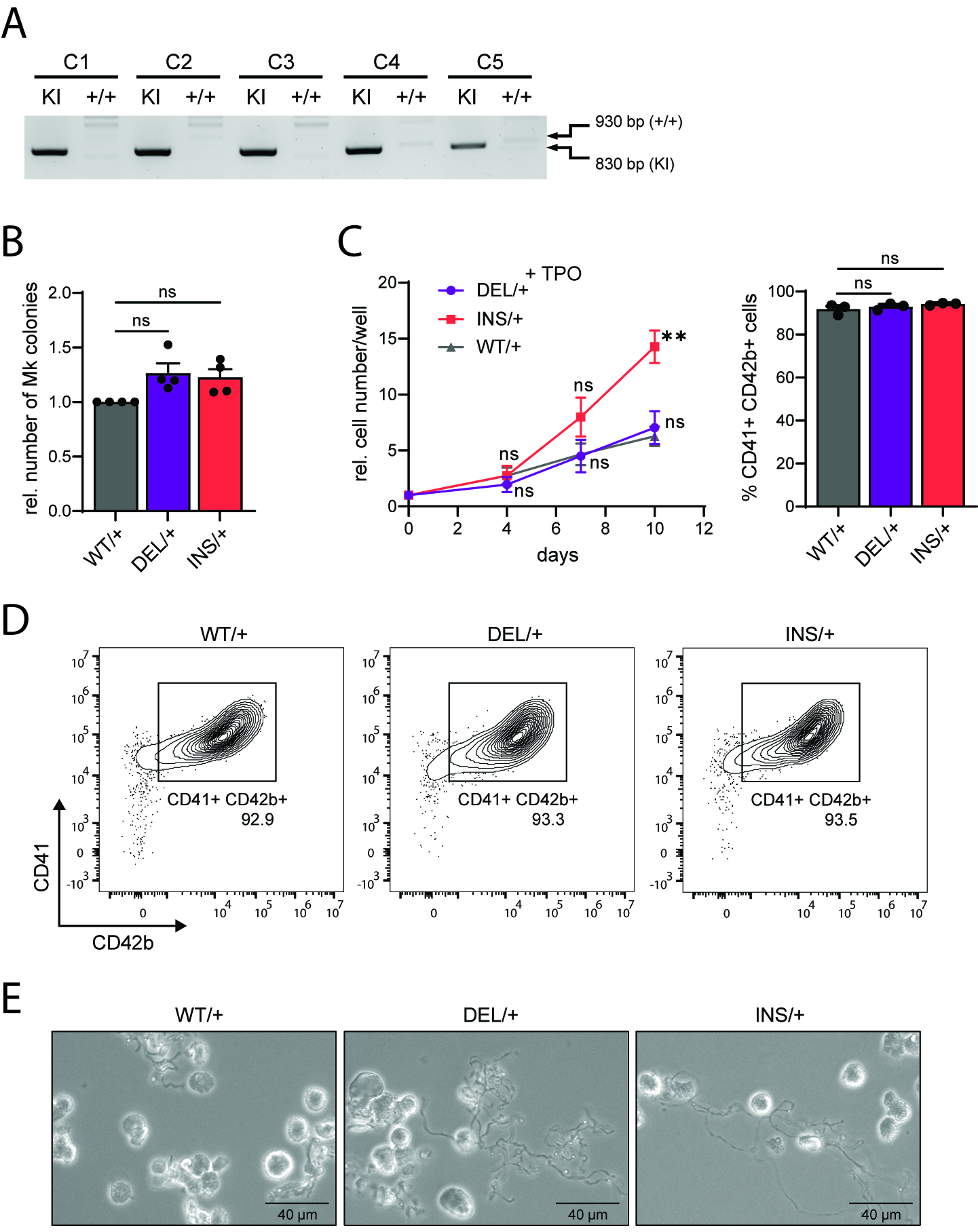
**

**Supplementary Figure 2. *CALR* mutations induce TPO-independent growth and megakaryocyte differentiation in HSPCs*.* (A)** Confirmation of *CALR* cDNA knock-in via ‘in-out’ PCR in five randomly picked colonies (C1-5) of all lineages from a methylcellulose-based CFU assay. Bi-allelic knock-in was confirmed by the presence of *CALR* cDNA (KI lanes) and absence of any non-integrated alleles (+/+ lanes). **(B)** Megakaryocyte specific CFU assay with bi-allelic engineered HSPCs in collagen-based TPO (50 ng/ml) containing media. N = 4 independent biological replicates. **(C)** Proliferation (left) and megakaryocytic differentiation (right) of mono-allelic engineered HSPCs in high TPO (50 ng/ml) liquid culture (three independent biological replicates). Megakaryocyte differentiation was assessed by CD41 and CD42b cell surface marker expression at d10 of culture. **(D)** Representative flow plots showing CD41 and CD42b megakaryocyte marker expression after 10 days of culture. **(E)** Light microscopy images of megakaryocytes in liquid culture forming protrusions to shed platelets, indicative of terminal differentiation.

**Supplementary Figure 3. Genome-engineered human *CALR* MUT HSPCs show robust engraftment and induce myelofibrosis and splenomegaly in mice. (A)** Representation of CRISPR-modified cell fraction within bulk of HSPCs from three independent cord blood donors prior to transplant. **(B)** Representative images of *CALR* MUT protein stained CRISPR-engineered cells isolated via FACS from the BM of engrafted primary recipient mice 24 weeks post-Tx. **(C)** Representative images of H&E (top) and hCD45 (bottom) stained spleen sections from NSG mice transplanted with either *CALR* MUT or WT HSPCs. Images with lower (left panel) and higher (right panel) magnification are shown to evaluate tissue and cell morphology. **(D)** Schematic workflow of competitive xenotransplantation experiments. 1x10^5^ sort-purified human CD34^+^ HSPCs with *CALR* MUT/+ or WT/+ knock-in were mixed at a 1:1 ratio and intrahepatically transplanted into newborn NSGW41 mice. Created with BioRender.com. **(E)** Percentage of modified cells within hCD45^+^ engrafted cells in competitive transplanted NSGW41 mice was evaluated 16 weeks post-Tx. N = 7 mice from one cord blood donor. **(F)** Representative flow plot showing percentages of *CALR* MUT (BFP) and WT (GFP) cells within pre-gated hCD45^+^ engrafted cells. **(G)** Schematic workflow of xenotransplantation experiments. 5x10^5^ human CD34^+^ HSPCs, containing a sub-fraction of cells with *CALR* MUT/+ or WT/+ knock-in, were intrafemorally transplanted into 8-12 week-old sub-lethally irradiated (100 rad) NSG mice. FACS-based enrichment prior to transplantation was avoided to reduce cellular stress. Consecutive BM aspirations were performed to evaluate human engraftment. Mice were sacrificed after 24 weeks, BM collected, and hCD45^+^ enriched cells transplanted into secondary recipient mice. Created with BioRender.com. **(H)** Percentages of total hCD45^+^ (left) and modified cells with engrafted hCD45^+^ cells (right) in the BM of secondary recipient mice were analyzed at 8 and 16 weeks post-Tx via flow cytometry. N = 5 mice from three independent cord blood donors.

**Supplementary Figure 4. *CALR* MUT human HSPCs show dysregulated ER stress response mechanisms*.* (A)** Volcano plot visualizing up- or down-regulated genes in *CALR* INS vs. DEL HSPCs. Significantly deregulated genes (p_adj_ < 0.05) are shown in black and genes with FC < 0.6 were additionally highlighted in red. **(B)** Heatmap showing the expression (z-score) of significantly DEGs in each sample and the clustering of samples based on their gene expression profile. **(C)** Differential expression of 10 selected DEGs detected by RNA-seq was confirmed via RT-qPCR in engineered *CALR* MUT (DEL, INS) and WT HSPCs from three independent cord blood donors. **(D-F)** Forest plots summarizing enriched terms from GSEA results using the Reactome gene set database. Enriched gene sets were ranked based on their normalized enrichment score. **(G)** Enrichment analysis (Enrichr webtool, maayanlab.cloud/Enrichr) of significantly down-regulated genes (padj < 0.05) comparing INS vs. DEL was performed, and the top 7 enriched terms from Reactome and Bioplanet gene set databases were reported.

**Supplementary Table 1. Summary of MPN patient clinical information**

Clinical information of all *CALR* MUT MPN patients, of which CD34^+^ HSPCs were used for RT-qPCR of selected genes, were listed here. Blood parameters were reported from time of diagnosis. *CALR* and additional co-mutations were detected via next-generation sequencing of peripheral blood derived cells. Dx = diagnosis, Hb = hemoglobin levels, Plt = platelet counts, WBC = white blood cell counts, PB blasts = peripheral blood CD34^+^ blasts, ET = essential thrombocythemia, PMF = primary myelofibrosis, p-ET MF = post-essential thrombocythemia myelofibrosis

| **Patient ID** | **Sample ID** | **Diagnosis (Dx)** | **Age at Dx** | **Sex** | **CALR mutation** | **Co-mutations** | **Spleen size (cm)** | **Hb (g/dl)** | **Plt (x10^9^/L)** | **WBC (x10^9^/L)** | **PB blasts (%)** |
| --- | --- | --- | --- | --- | --- | --- | --- | --- | --- | --- | --- |
| LB-MUG-035 | 9703 | PMF | 55.2 | F | c.1099_1150del  (p.L367fs*46) | - | 22 | 11.2 | 341 | 8.98 | 3 |
| LB-MUG-010 | 9793 | p-ET MF | 40.7 | F | c.1099_1150del (p.L367fs*46) | NRAS c.G35A  (p.G12D), ASXL1  c.2467delT (p.L823*) | 20 | 12.2 | 437 | 12.06 | 1 |
| LB-MUG-004 | 9800 | p-ET MF | 60.2 | F | c.1154_1155insTTGTC  (p.K385fs*47) | ASXL1 c.1772dupA  (p.Y591*), PTPN11  c.A922G (p.N308D) | N/A | 10.0 | 321 | 6.65 | 1 |
| LB-MUG-332 | 10049 | p-ET MF | 63.4 | F | c.1154_1155insTTGTC  (p.K385fs*47) | ASXL1 c.1772dupA  (p.Y591_Q592delinsX) | 28 | 11.0 | 724 | 14.13 | 1 |
| LB-MUG-230 | 10059 | PMF | 74.5 | M | c.1099_1150del  (p.L367fs*46) | SF3B1 c.G1998T  (p.K666N) | 25 | 11.3 | 253 | 12.60 | 1 |
| LB-MUG-005 | 9549 | PMF | 29.0 | M | c.1099_1150del  (p.L367fs*46) | MPL c.T1543A  (p.W515R) | 13 | 14.1 | 516 | 10.02 | 1 |
| LB-MUG-372 | 9567 | ET | 40.4 | F | c.1154_1155insTTGTC  (p.K385fs*47) | N/A | 12 | 14.2 | 1557 | 10.33 | 1 |
| LB-MUG-338 | 9731 | ET | 50.0 | M | 43 bp deletion | N/A | N/A | 13.4 | 1136 | 7.00 | 1 |
| LB-MUG-067 | 9581 | PMF | 64.6 | F | c.1119_1139delinsGC TTTGCGTTTCTT (p.D373_E380delinsEL CVSWfs) | NRAS c.T190A  (p.Y64N) | 18 | 12.6 | 390 | 17.81 | 3 |
| LB-MUG-159 | 9910 | p-ET MF | 58.8 | F | c.1099_1150del (p.L367fs*46) | - | N/A | 7.9 | 319 | 4.78 | 2 |

# Supplementary Methods

**Primary cell isolation**

Umbilical cord blood (UCB) was collected from term deliveries at the Department of Obstetrics and Gynecology, Medical University of Graz following informed consent and institutional review board approval (IRB approval: 31-322 ex 18/19). After enriching mononuclear cells (MNC) from whole blood by density gradient separation (Ficoll Paque Plus, GE Healthcare; 400g, room temperature, 30 minutes, deceleration off), primary CD34^+^ HSPCs were purified via magnetic bead-based positive selection (CD34 MicroBead, Miltenyi Biotech, Bergisch Gladbach, Germany) according to the manufacturer’s protocol. CD34^+^ cells with a purity above 90 % were cryopreserved for long-term storage. Peripheral blood (PB) from ET, PMF and post-ET MF patients was collected at the Division of Hematology, Medical University of Graz following informed consent and institutional review board approval (IRB approval: 30-464 ex 17/18). MNCs were enriched from fresh whole blood by density gradient separation and stored in liquid nitrogen upon use. For experiments, MNCs were thawed and Lin^-^ CD34^+^ cells purified via FACS prior to culture.

**Cell culture**

Purified CD34^+^ cells were cultured in serum-free stem cell retention media (StemSpan SFEMII, Stemcell Technologies, Vancouver, BC, Canada) supplemented with human recombinant SCF, TPO, FLT3, IL-6 (all 100 ng/ml), UM171 (35 nM) and StemReginin 1 (SR1, 750 nM). All cytokines and SR1 were purchased from PeproTech (Rocky Hill, NJ, USA) whereas UM171 was purchased from Stemcell Technologies. TF-1_TpoR cells were generally cultured in RPMI1640 (Sigma Aldrich, St. Louis, MO, USA) supplemented with 10% fetal bovine serum (FBS; PAN-Biotech, Aidenbach, Germany) and 10 ng/ml TPO, whereas TPO was removed for TPO-independent cultures of engineered *CALR* mutant cells. Parental TF-1 cells were purchased from ATCC (Manassas, VA, USA) and tested for identity by STR profiling.

**Single guide RNA (sgRNA) design**

The *CALR* sgRNA was designed to target intron 7 upstream of the mutational hotspots in exon 9. The target sequence (with PAM in bold) was: 5’-CGCCTGTAATCCTCGCCCAG**AGG**-3’; All sgRNAs were acquired from Synthego (Redwood City, CA, USA) as chemically modified sgRNAs containing 2'-O-Methyl groups at the three first and last bases and 3'-Phosphorothioate bonds between the first 3 and the last 2 bases.

**AAV vector production**

AAV vector plasmids were cloned in the pAAV-MCS plasmid (#240071, Agilent, Santa Clara, CA, USA) containing ITRs from AAV serotype 2 (AAV2). Our *CALR* targeting vector contained a codon optimized *CALR* cDNA spanning exons 8 and 9 with either wildtype (WT) sequence or harboring a type 1 (52 bp deletion) or type 2 (5 bp insertion) *CALR* mutation followed by a SV40 poly-adenylation signal. An upstream (5’) splice acceptor (150 bp) was added to ensure proper splicing of the integrated cDNA to the endogenous exons 1-7. Downstream (3’) of the cDNA construct we additionally included a fluorescent reporter gene (GFP or BFP) under control of an SFFV-promoter, to allow for enrichment of successfully modified cells. WT cDNA was combined with GFP and MUT cDNA with BFP, enabling recognition of the respective genotypes (homozygous or heterozygous) by color. The left and right homology arms were 250 bp and 400 bp respectively. rAAV6 vectors were produced as described previously.^1^ Briefly, 293FT cells (Life Technologies, Carlsbad, CA, USA) were seeded at 13x10^6^ cells per dish in ten 15-cm dishes one day before transfection. Each 15-cm dish was transfected using standard PEI transfection with 6 μg ITR-containing plasmid and 22 μg pDGM6 (#110660, Addgene, Watertown, MA, USA), containing AAV6 cap genes, AAV2 rep genes, and adenovirus 5 helper genes. Cells were incubated for 72 hours until rAAV6 was purified using the AAVpro^®^ Purification Kit (Takara Bio, Shiga, Japan) following the manufacturer’s recommendations. Viral vectors were aliquoted and stored at -80°C until further use. Number of vector genomes within the rAAV6 preps was quantified by ddPCR (QX200, Bio-Rad, Hercules, CA, USA).

**Electroporation and transduction of cells**

Cas9 protein and sgRNAs were complexed at a molar ratio of 1:2.5 at 25˚C for 10 min to form Cas9 RNP immediately before nucleofection into CD34^+^ HSPCs. Cas9 protein was purchased from Integrated DNA Technologies (IDT, Coralville, IA, USA). CD34^+^ HSPCs were electroporated with 230 µg/ml RNP complex 72 hours after culture initiation using the Lonza 4D Nucleofection system (program DZ-100) and the Human Primary Cell Nucleofection Kit P3 (Lonza, Basel, Switzerland). Following electroporation, cells were transferred into fresh culture media and immediately transduced with AAV6 donor vectors at an MOI (vector genomes/cell) of 5,000-10,000. Cell culture media was replaced after 6-8 hours to reduce viral exposure. 48 hours after nucleofection and transduction, cells with high reporter expression (GFP, BFP or mCherry), indicative of successful CRISPR/Cas9-mediated recombination were enriched on an Aria IIIu cell sorter (BD Biosciences, San Jose, CA, USA).

**Confirmation of site-specific knock-in**

For DNA extraction, 5x10^4^ sort purified cells or picked, single colonies from CFU assays were suspended in 25 µl and 10 µl QuickExtract DNA Extraction Solution (Epicentre, Madison, WI, USA) respectively, and processed according to manufacturer’s recommendations. Site-specific integration was detected by ‘in-out’ PCR using a primer that binds outside the homology arm (HA) and a primer specific for the transgene cassette (insert). *CALR*_outside_LHA: 5’-AAGTGATCCGTTCGCCATGAC-3’, *CALR*_WT_insert: 5’-ACGTCCTCTTCCTCGTCCTC-3’, *CALR*_DEL_insert: 5’-CCAACCCTGGAGACACGCTTC-3’, *CALR*_INS_insert: 5’-TCAGTCCATCCCTGCAAGCAC-3’, All integrated PCR bands were subjected to Sanger sequencing to confirm correct and seamless integration at the intended locus.

**Immunocytochemical staining of *CALR* MUT**

5x10^4^ sort-purified CRISPR-modified primary human cells were spun onto Super Frost Plus™ glass slides (Thermo Fisher Scientific, Waltham, MA, USA) and air dried for 30 minutes. Cells were fixed with absolute methanol for 5 minutes, permeabilized with PBS + 0.1% Triton X-100 for 5 minutes and washed with PBS + 0.01% Tween-20. Subsequently, slides were blocked with 5% horse serum for 1 hour and afterwards labeled with 1:100 diluted mouse anti-mutant *CALR* (CAL2, dianova, Hamburg, Germany) primary antibody for another hour at 37°C in a humidified chamber. Slides were washed three times, labeled with ImmPRESS anti-mouse HRP secondary antibody for 1 hour at 37°C and washed again. Final staining of cells was performed using a DAB HRP substrate (Vector Laboratories, Burlingame, CA, USA). Cell nuclei were counter-stained with DAPI. Cells were evaluated for *CALR* MUT protein expression on a standard light microscope (Olympus BX51).

**Immunoblotting**

CD34^+^ and TF-1_TpoR cells were lysed in cold RIPA buffer (Thermo Fisher Scientific, Waltham, MA, USA) supplemented with protease and phosphatase inhibitor cocktail (Thermo Fisher Scientific) by sonication for 3-5 minutes. Lysed samples were boiled at 95°C for 5 min after addition of 4x Laemmli buffer (Bio-Rad, Hercules, CA, USA) containing 10% β-mercaptoethanol. 30 µg of total protein lysate were loaded onto SDS gels for electrophoresis and transferred onto PVDF membranes (Bio-Rad). Primary antibodies against CALR (#12238), β -Actin (#4970), p-STAT1 (#9167), p-STAT3 (#9145), p-AKT (#4060), p-ERK1/2 (#4370), STAT1 (#9172), STAT3 (#4904), STAT5 (#94205), AKT (#4691), and ERK1/2 (#9102) were purchased from Cell Signaling Technology (Danvers, MA, USA). Antibodies against p-STAT5 (#611965) were purchased from BD Biosciences, against Vinculin (#ab129002) from Abcam (Cambridge, UK) and against mutant CALR (#DIA-CAL-250) from Dianova. All primary antibodies were used at 1:1000 dilutions. Secondary HRP-linked antibodies against rabbit (#7074) and mouse (#7076) were purchased from Cell Signaling Technology and used at 1:5000 dilutions.

**Methylcellulose-based colony forming assay (MethoCult™)**

Fluorescent reporter positive HSPCs were sorted into serum-free expansion media (SFEMII), admixed with semi-solid methylcellulose media (MethoCult™ Enriched, StemCell Technologies, #H4435) and seeded in triplicates into 35 mm dishes (300 cells per dish). Cells were incubated for 14 days at 37°C before developed colonies were counted and scored based on morphologic evaluation (CFU-GM: colony-forming unit granulocyte/macrophage, CFU-M: colony-forming unit monocyte, BFU-E: burst-forming unit erythroid) according to the manual “Human CFU assays using MethoCult” from StemCell Technologies.

**Collagen-based megakaryocyte colony forming assay (MegaCult™)**

Fluorescent reporter positive HSPCs were sorted into MegaCult™-C medium with lipids (StemCell Technologies) containing 10 ng/ml IL-3 and IL-6 and 50 ng/ml TPO. To evaluate TPO-independent colony outgrowth, cytokine containing media without TPO was used. Suspended cells were mixed with collagen prior to seeding in quadruplicates into 2-well chamber slides (1800 cells per well) and incubated for 12 days at 37°C. Collagen embedded colonies were fixed on the slides and stained for CD41-expression using a primary mouse anti-CD41 or an isotype control antibody (Biolegend, San Diego, CA, USA) followed by an anti-mouse alkaline phosphatase (AP) secondary antibody (Vector Laboratories, Burlingame, CA, USA). To visualize megakaryocyte (CD41 positive) colonies, magenta staining was applied using AP substrate Vector^®^ Red. Non-megakaryocytic (CD41 negative) colonies appeared only blue due to Evans blue counter-staining. Colonies were distinguished and counted based on their staining.

**Megakaryocyte liquid culture differentiation**

Fluorescent reporter positive HSPCs were sorted into StemSpan SFEMII (Stemcell Technologies, Vancouver, BC, Canada) supplemented with 1 ng/ml SCF, 5 ng/ml TPO, 10 ng/ml IL-6, 10 ng/ml IL-9 and 0.4% LDL and plated into 48-well plates. For each time point, cells were seeded into a separate well to minimize manipulation. On day 7, half of the culture media was replaced with fresh growth media containing cytokines. On days 4, 7, and 10, cells were stained with CD41-APC-Cy7 and CD42b-APC (BioLegend, San Diego, CA, USA) fluorochrome conjugated antibodies. Counting beads (CountBright, Thermo Fisher Scientific, Waltham, MA, USA) were added to the cells prior to measurements on the flow cytometer (CytoFLEX S, Beckman Coulter, Brea, CA, USA) to analyze the differentiation and the cell number simultaneously. Flow data was analyzed using FlowJo v10.8 (FlowJo LLC, Ashland, OR, USA). Early megakaryocytic progenitors were defined as CD41^+^ CD42b^-^, and mature platelet-forming megakaryocytes as CD41^+^ CD42b^+^.

**Xenotransplantation of CD34+ human HSPCs into immune-compromised mice**

All animal experiments were conducted in accordance with a protocol of the Institutional Animal Care and Use Committee at Medical University of Graz, Austria and approved by the Austrian Ministry for Science (GZ: BMWFW-66.010/0018-V/3b/2018). FELASA guidelines to minimize animal distress and suffering were strictly followed. NSG breeder mice were purchased from Jackson laboratories (Bar Harbor, ME, USA). Mice were bred in house and housed in individually ventilated cages under specific pathogen free (SPF) conditions. Due to the lack of preliminary data about the engraftment of our genome-engineered HSPCs, no specific sample size calculation could be performed. Furthermore, mice were not randomized and investigators were not blinded for the following procedures. 8 to 12 week old female NSG mice were irradiated (100 rad, RS2000 x-ray Irradiator, Rad Source Technologies, Buford, GA, USA) 24 hours prior to transplantation. A total of 5x10^5^ HSPCs, containing a mix of reporter positive (CRISPR-modified) and reporter negative (unmodified) HSPCs were intrafemorally (if) transplanted into irradiated NSG mice, 8 hours after nucleofection and AAV6-transduction (n=2 mice each genotype and donor, cells from three independent UCB donors). For competitive transplants, reporter positive (CRISPR-modified) human HSPCs were sort-purified 48 hours post modification and 1x10^5^ of *CALR* MUT and WT cells mixed in a 1:1 ratio were intrahepatically transplanted into 48 hours old new-born NSGW41 mice (n=7, cells from one UCB donor). Short-term (8 weeks) and long-term (16 and 24 weeks) engraftment of human cells in the murine BM was assessed via flow cytometry. After RBC lysis (RBC lysis buffer, eBioscience, San Diego, CA, USA), cells were stained with fluorochrome conjugated monoclonal antibodies: 1:50 dilution of mTer119-BUV661 (TER-119), mCD45-APC-Cy7 (30-F11), hCD45-BB700 (HI30), CD33-PE (WM53, BD Biosciences, San Jose, CA, USA), CD19-SB600 (SJ25C1, eBioscience, San Diego, CA, USA), CD41-PE-Cy7 (HIP8, BioLegend, San Diego, CA, USA) and for viability using a 1:1000 dilution of SYTOX Red (Invitrogen, Carlsbad, CA, USA). Measurement was performed on a 5-laser flow cytometer (CytoFLEX LX, Beckman Coulter, Brea, CA, USA) and post-hoc analyses were performed using FlowJo v10.8 (FlowJo LLC, Ashland, OR, USA).

**RNA sequencing**

2x10^5^ fluorescent reporter positive, CRISPR-modified (heterozygous *CALR* MUT or WT) CD34^+^ human cord blood derived HSPCs (3 individual donors) were sort-purified (BD FACS Aria IIIu) and cultured for two more days in stem cell retention media before isolation of RNA (Monarch Total RNA Miniprep Kit, NEB, Ipswich, MA, USA). RNA concentration was determined using the Qubit 2.0 Fluorometric Quantitation system (Thermo Fisher Scientific, Waltham, MA, USA) and RNA quality was assessed using the Experion Automated Electrophoresis System (Bio-Rad, Hercules, CA, USA). RNA-seq libraries were prepared with the TruSeq Stranded mRNA LT sample preparation kit (Illumina, San Diego, CA, USA) using Sciclone and Zephyr liquid handling workstations (PerkinElmer, Waltham, MA, USA) for pre- and post-PCR steps, respectively. Library concentrations were quantified via Qubit and the size distribution was assessed using the Experion Automated Electrophoresis System. For sequencing, samples were diluted and pooled into NGS libraries in equimolar amounts. Libraries were sequenced on a HiSeq 3000 instrument (Illumina, San Diego, CA, USA) performing 50-base-pair, single-end sequencing. The raw sequencing data have been deposited in NCBI's Gene Expression Omnibus (Foßelteder et al., 2022) and are accessible through GEO Series accession number GSE195705 (<https://www.ncbi.nlm.nih.gov/geo/query/acc.cgi?acc=GSE195705>). NGS reads were mapped to the Genome Reference Consortium GRCh38 assembly via “Spliced Transcripts Alignment to a Reference” (STAR) utilizing the “basic” Ensembl transcript annotation from version e100 (April 2020) as reference transcriptome. Aligned NGS reads overlapping Ensembl transcript features were counted with the Bioconductor (v3.12) GenomicAlignments (v1.26.0) package via the summarizeOverlaps function in Union mode. Transcript-level counts were aggregated to gene-level counts and the Bioconductor DESeq2 (v1.30.0) package was used to test for differential expression based on a model using the negative binomial distribution. Volcano plots and heatmaps were generated using the Bioconductor packages ggplot2 (3.3.3), EnhancedVolcano (1.8.0) and ComplexHeatmap (2.6.2). Gene set enrichment analysis (GSEA) was based on normalized read counts from the DESeq2 package (GSEA v.4.1.0, BROAD Institute, Cambridge, MA, USA) and gene sets were derived from the Molecular Signatures Database (MSigDB 7.4). Only gene sets with false discovery rate (FDR) < 0.25 were considered and normalized enrichment score (NES) was reported along the enrichment plots. Significantly differentially expressed genes (DEG) with padj < 0.05 from the DESeq2 package were used for pathway enrichment analysis using the Enrichr webtool (maayanlab.cloud/Enrichr). Enriched terms were selected based on the highest combined score (log p-value from Fisher exact test multiplied with the z-score of the deviation from the expected rank) from the Bioplanet 2019 and Reactome 2016 gene set databases. The top 10 common DEGs from DEL vs. WT and INS vs. WT were used for a STRING protein interaction network analysis (STRING v11.5, Elixir, Hinxton, Cambridgeshire, UK). Only interactions with high evidence (interaction score > 0.7) were plotted with max. 10 interaction partners per protein. Proteins were clustered via MCL with an inflation parameter of 3.

**RT-qPCR**

To confirm DEGs identified in the RNA-seq experiments, total RNA from CRISPR-modified HSPC of three independent cord blood donors was extracted (Monarch Total RNA Miniprep Kit, NEB) according to manufacturer’s protocol. Reverse transcription of RNA was performed using the LunaScript RT SuperMix Kit (NEB). Quantitative PCR was performed using a dye-based Luna Universal qPCR Master Mix (NEB). Gene-specific sequences were amplified on a QuantStudio 5 Real-Time PCR system (Applied Biosystems) using the following primers: *PRKCZ* fwd 5’- CAACTGCAAACTGCTGGTCC-3’, rev 5’-AGGCATGACAGAATCCATATGCT-3’, *CRELD2* fwd 5’-CAAGTACGAGTCCAGCGAGAT-3’, rev 5’- TGGAGAGCAGCACACTTTCA-3’, *MANF* fwd 5’- GTCACATTCTCACCAGCCACT-3’, rev 5’-GGCCCCGATATAGTAGCACAAC-3’, *HSPA5* fwd 5’- CATCAACGAGCCTACGGCA-3’, rev 5’-AGACACATCGAAGGTTCCGC-3’, *PDIA3* fwd 5’-CACGGACGACAACTTCGAGA-3’, rev 5’-TGGCAGTGCAATCAACCTTTG-3’, *PDIA4* fwd 5’-CTCCAGAACCCAGGAAGAAATTG-3’, rev 5’-TTCTCATACTCGGGGGCAAG-3’, *HSP90B1* fwd 5’-CCAGCAGAAAAGAGGCTGAAT-3’, rev 5’-ATTCGGGAAGGGCCTGAATAC-3’, *DNAJB11* fwd 5’-GGTGCTGCTTATGAGGTTCTGT-3’, rev 5’-GTCTTGCTGACGAGGGGTTC-3’, *HYOU1* fwd 5’-AACCTGAGAAAGTAGAGACTGGA-3’, rev 5’-CTTTCTGTTCAGGTTCTGCTCC-3’, SDF2L1 fwd 5’-TGTCCAACAACCAGGAGGTGAG-3’, rev 5’-AGCAGCGCACTGTCCATAG-3’, *CALR* fwd 5’-GATCCCACAGACTCCAAGCC-3’, rev 5’-GGCTTCCACTCACCCTTGTA-3’,
*HPRT1* fwd 5’- TGAGGATTTGGAAAGGGTGT-3’, rev 5’- GAGCACACAGAGGGCTACAA-3’, *GUSB* fwd 5’-GAAAATATGTGGTTGGAGAGCTCATT-3’, rev 5’-CCGAGTGAAGATCCCCTTTTTA-3’, *GAPDH* fwd 5’-GTCTCCTCTGACTTCAACAGCG-3’, rev 5’-ACCACCCTGTTGCTGTAGCCAA-3’

All genes were measured in technical duplicates and *HPRT1*, *GUSB*, and *GAPDH* were used as housekeeping genes (HKG). Gene expression in DEL and INS samples was normalized the three HKGs and to WT samples via the ΔΔCT method and mean fold changes were reported.

**Inhibitor treatments**

Sort-purified HSPCs and pre-diluted inhibitors were directly mixed with methylcellulose media (MethoCult™ Enriched) before seeding in triplicates (300 cells each) into 6-well SmartDishes™ (Stemcell Technologies) or with collagen-based media (MegaCult™-C) with 50 ng/ml TPO before seeding in quadruplicates (1800 cells each) into 2-well chamber slides (Thermo Fisher Scientific). MethoCult™ colonies were automatically counted after 14 days using the STEMvision™ device (Stemcell Technologies). MegaCult™ colonies were fixed after 12 days and stained for CD41 via immunocytochemistry before counting. Colony counts of inhibitor treated samples were normalized to a respective DMSO treatment control. Bortezomib and HA15 were dissolved and diluted in DMSO, and final concentrations were 2 nM and 5 µM respectively. Both inhibitors were purchased from MedChemExpress (Monmouth Junction, NJ, USA).

**Statistical analyses**

All statistical analyses were performed using Graphpad PRISM 9 (Graphpad Software, San Diego, CA, USA). One-way ANOVA with Dunnett’s multiple comparison correction was used to test for statistical significance between *CALR* MUT and WT samples in bar graphs of *in vitro* experiments. Two-way ANOVA with Dunnett’s multiple comparison correction was used in line graphs of TF-1_TpoR cultures and megakaryocyte differentiation to test for differences at several time points. Statistical significance of *in vivo* studies was either evaluated using two-way ANOVA with Sidak’s multiple comparison correction for human engraftment analysis at subsequent time points or using an un-paired student’s t-test (two-sided) with Welch’s correction for single time point analyses. All data was checked for normal distribution prior to statistical testing. If similarity of variances between groups was not given, Geisser-Greenhouse correction was used. P-values of less than 0.05 were considered statistically significant and the degree of significance was reported using asterisks (* p < 0.05, ** p < 0.01, *** p < 0.001). Summarized data was reported with mean ± standard error of mean (SEM) of independent biological replicates in bar and line graphs.

**Data availability statement**

RNA-Seq data are available at the GEO repository under accession number GSE195705. Original data generated in this study is available from the corresponding author on request.

**References**

1. Bak RO, Dever DP, Porteus MH. CRISPR/Cas9 genome editing in human hematopoietic stem cells. *Nature Protocols*. 2018;13(2):358-376.
